# Post-COVID syndrome screening through breath analysis using electronic nose technology

**DOI:** 10.1007/s00216-022-03990-z

**Published:** 2022-03-18

**Authors:** Nidheesh V. R., Aswini Kumar Mohapatra, Unnikrishnan V. K., Jijo Lukose, Vasudevan Baskaran Kartha, Santhosh Chidangil

**Affiliations:** 1 Centre of Excellence for Biophotonics, Department of Atomic and Molecular Physics, Manipal Academy of Higher Education, Manipal, Karnataka India 576104; 2 Department of Respiratory Medicine, Kasturba Medical College, Manipal, Manipal Academy of Higher Education, Manipal, Karnataka India 576104

**Keywords:** Post-COVID syndrome, Breath analysis, E-nose, Biosensor, Chemometrics

## Abstract

There is an urgent need to have reliable technologies to diagnose post-coronavirus disease syndrome (PCS), as the number of people affected by COVID-19 and related complications is increasing worldwide. Considering the amount of risks associated with the two chronic lung diseases, asthma and chronic obstructive pulmonary disease (COPD), there is an immediate requirement for a screening method for PCS, which also produce symptoms similar to these conditions, especially since very often, many COVID-19 cases remain undetected because a good share of such patients is asymptomatic. Breath analysis techniques are getting attention since they are highly non-invasive methods for disease diagnosis, can be implemented easily for point-of-care applications even in primary health care centres. Electronic (E-) nose technology is coming up with better reliability, ease of operation, and affordability to all, and it can generate signatures of volatile organic compounds (VOCs) in exhaled breath as markers of diseases. The present report is an outcome of a pilot study using an E-nose device on breath samples of cohorts of PCS, asthma, and normal (control) subjects. Match/no-match and *k*-NN analysis tests have been carried out to confirm the diagnosis of PCS. The prediction model has given 100% sensitivity and specificity. Receiver operating characteristics (ROC) has been plotted for the prediction model, and the area under the curve (AUC) is obtained as 1. The E-nose technique is found to be working well for PCS diagnosis. Our study suggests that the breath analysis using E-nose can be used as a point-of-care diagnosis of PCS.

**Trial registration**

Breath samples were collected from the Kasturba Hospital, Manipal. Ethical clearance was obtained from the Institutional Ethics Committee, Kasturba Medical College, Manipal (IEC 60/2021, 13/01/2021) and Indian Council of Medical Research (ICMR) (CTRI/2021/02/031357, 06/02/2021) Government of India; trials were prospectively registered.

**Graphical abstract:**

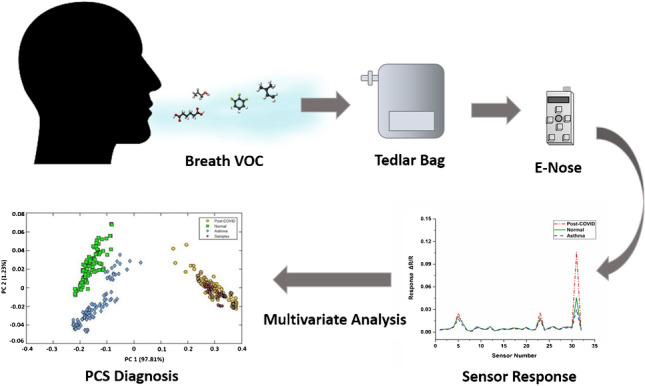

**Supplementary Information:**

The online version contains supplementary material available at 10.1007/s00216-022-03990-z.

## Introduction

With the number of people affected by COVID-19 infection increasing steadily, especially with repeated waves of infection with newly diagnosed variants, post-coronavirus disease syndrome (PCS) instances have also started increasing, with large numbers of deaths due to various complications associated with the respiratory system from PCS. PCS is the wide range of new, returning, or ongoing health problems one can experience after four or more weeks of being infected by COVID-19 [1]. An observational cohort study that evaluated the outcomes of 1250 patients discharged at the 60th day has found that 6.7% of patients died during the study period, and 15.1% of patients required re-admission. Moreover, 32.6% of patients reported persistent symptoms, which included 18.9% with new or worsened symptoms [2]. PCS symptoms include difficulty in breathing, chest pain, fatigue, sleep issues, brain fog, including an inability to concentrate and impaired memory, and loss of taste and/or smell [3]. Many PCS-related respiratory discomforts are similar to those observed with lung diseases like asthma and COPD. PCS is thus, not clearly distinguishable from manifestations that occur after asthma, COPD, and many other acute viral diseases and after prolonged stays in ICUs due to other diseases [4]. More importantly, continued assessment of PCS patients is essential because such long-term health consequences, even after very mild COVID-19, may pose serious medical, social, and economic challenges [5]. Also, it has been observed that PCS often leads to multi-organ damage which affects many systems in the body, including heart, lung, kidney, blood vessels, and brain, with phenomena like multisystem inflammatory syndrome (MIS), a condition where different organs become severely inflamed, with signs and symptoms varying, depending on which organ/area is affected. A sharp spike in multi-organ inflammatory syndrome in children (MIS-C) has also been observed [6]. MIS-C cases are typically reported 3–6 weeks after the COVID-19 infection, 84% of cases show positive for SAR-CoV-2. All cases have a history of COVID-19; therefore, there is strong evidence that the worst cases of PCS turn into MIS-C as an aftereffect of COVID-19 [7]. The rise in the numbers of MIS-C, mostly between 1 and 18 years, along with rare cases in 6-month-old babies, is a cause for great worry. Many of the PCS symptoms are seen even after several months, up to even 7 months. It is not known at present how long the multi-organ system effects might last and whether the effects could lead to chronic health conditions [8].

Present methods to detect PCS symptoms include MRI, blood tests, chest X-ray, and echocardiography of the heart [6]. All these methods are rather difficult to pursue in many situations, especially in cases of MIS-C or older adults. There is thus an urgent need for reliable screening methods to discriminate PCS from conditions that produce similar symptoms. Any deviation from the normal functioning of the living system leads to various bio-molecular interactions, which produce many markers specific to the condition. These markers include many volatile organic compounds (VOCs), which will be transported by circulating blood to the lungs to be eventually discharged in the exhaled breath. Breath analysis is thus getting a lot of attention for diagnostic applications due to its totally non-invasive nature and ease of use even in difficult situations like subjects in ICU, infants, and geriatric-condition patients. There are different methods available for breath analysis at present, including laser spectroscopy, gas chromatography (GC), gas chromatography–mass spectrometry (GC–MS), and electronic (E-)nose technology [9]. Laser spectroscopy and GC–MS can provide individual molecular identification. But these techniques are complex, require costly equipment, and are time-consuming, while the electronic nose can be used for point-of-care applications due to instrument size, cost, and immediate results, once standardized. Present E-nose technology involves nano-composite gas sensor arrays, metal oxide semiconductors, or quartz crystal microbalance [10, 11]. Snitz et al. [12] have proven the ability of E-nose technology for the rapid detection of COVID-19. The E-nose device used in the present study has carbon nano-composite gas sensor arrays which work on the principle of chemi-resistive technique, where the resistance of the sensor changes in accordance with the interaction of the VOCs with the sensors. Cyranose-320, a sensor array with 32 sensors, has been used by different groups to diagnose and classify different lung diseases such as COPD, asthma, lung cancer, malignant mesothelioma, and common cold [9, 13–16]. In a recently reported publication by Zamora-Mendoza et al. [17], breath analysis of COVID, PCS, and normal samples used Cyranose-320 with a sensitivity of 97.4% in the classification among PCS and normal samples. The breath analysis method can be successfully used for point-of-care applications in diagnosing PCS. Non-invasive, cost-effective, need of less expertise, and fast diagnosis (result in hand within 10 min) are the advantages of this technique over other usual clinical procedures. Below we discuss the discrimination and classification of PCS, asthma, and normal breath samples using the E-nose technology, combined with multivariate analysis.

## Methods

### Study design and participants

Breath samples were collected from the Kasturba Hospital, Manipal. Ethical clearance was obtained from the Institutional Ethics Committee (IEC 60/2021) and ICMR (CTRI/2021/02/031357), Government of India. Twenty-four each PCS and normal volunteers are involved in the study. All the PCS volunteers were subjects treated at Kasturba Hospital for COVID-19 positive and discharged after recovery. It was observed that after discharge, they were getting some respiratory problems like breathing difficulties and cough. All asthma patient volunteers were from the Department of Respiratory Medicine, Kasturba Hospital (with medical records), who have undergone NO breath test and confirmed NO positive with more than 40 ppb concentration and who are now under medication. Normal volunteers are healthy individuals with no COVID-19 history. Volunteers’ information sheet was given to all the participants, and samples were collected with informed consent. The participant’s habits and history are noted (Table S1).

### Procedure

**Fig. 1:**
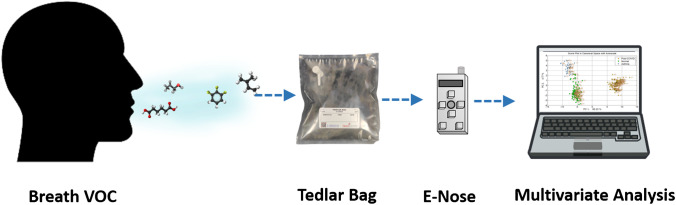
Schematic of the experimental procedure for breath VOC analysis

Breath samples were collected and stored in 3 l Tedlar gas sampling bags with integrated valves ^18,19^. To minimise the effects of room-air contaminants, subjects inhaled pure air using a VOC filter cartridge for 5 min. Two or three long exhaled breath of air was then collected. It was ensured that the subjects had not consumed any food for at least 4 h before sample collection. The investigators used personal protective equipment such as gloves, gown, laboratory coat, face shield, and mask while collecting and recording the sample. After each observation, the device was properly disinfected, and sampling bags were disinfected and disposed of away. The experimental procedure for the VOC analysis is shown in Fig. 1. Sample collection and recording have been made in ambient conditions with a temperature of 25 °C and humidity of 55%.

An ambient air purge of 60 s was carried out to create the baseline, followed by 40 s of sample draw. Data from all 32 sensing elements of the sensors have been taken for analysis. Parameter settings of the sensor, Cyranose-320, are shown in Table 1.

**Table 1:** Parameter settings for the Cyranose-320 sensor studies

Items	Time	Pump speed
Baseline purge	60 s	Medium
Sample draw	40 s	Medium
Sample draw 2	0	N/A
Snout removal	0	N/A
1st sample gas purge	0	N/A
1st air intake purge	60 s	High
2nd air intake purge	0	N/A
Digital filter ON substrate temperature 42 °C		
Algorithm	Canonical	
Pre-processing	Autoscaling	

### Data analysis

Data pre-processing was carried out in PC Nose + software (Sensigent). Method file was saved by connecting the E-nose to PC Nose + software for multivariate analysis. The average response from all 32 sensors, representing the Δ*R*/*R* values and score plot, has been plotted using CD analysis software. The *k*-nearest neighbours (k-NN) algorithm was used to assess the sensor’s performance to discriminate PCS, asthma, and normal breath samples using CD analysis software. For *k*-NN analysis, a reference data set is used and the distance between the recorded data and the reference data is calculated. In a *k*-NN model created with optimum *k* values, the recorded data will look at the *k*-closest value in the reference data set and predict the class based on the highest similarity. The model has been built for 5ks, and a prediction test for test samples of PCS data has been conducted ^20^.

Match/no-match test is a statistics tool where the test samples are compared with a standard “calibration” set. A standard calibration set of 100 PCS data (20 subjects) has been created using the GRAMS IQ option of GRAMS AI software (Thermo Scientific Inc., Rockford, IL). Test data of 5 each from four subjects of normal, asthma, and PCS was used for the match/no-match test in GRAMS IQ Predict. Score test, residual test, and Mahalanobis distance (M. distance) test are used for the match/no-match analysis. Calculation of sensitivity and specificity has been carried out using math/no-match results. Receiver operating characteristic (ROC) has been plotted using sensitivity and specificity values based on the cut-off thresholds of M. distances. The area under the curve (AUC) of ROC is the performance measurement for the classification. AUC-ROC value can vary from 0 to 1; the higher the AUC, the better the model will distinguish between the different classes of samples.

## Results

**Fig. 2:**
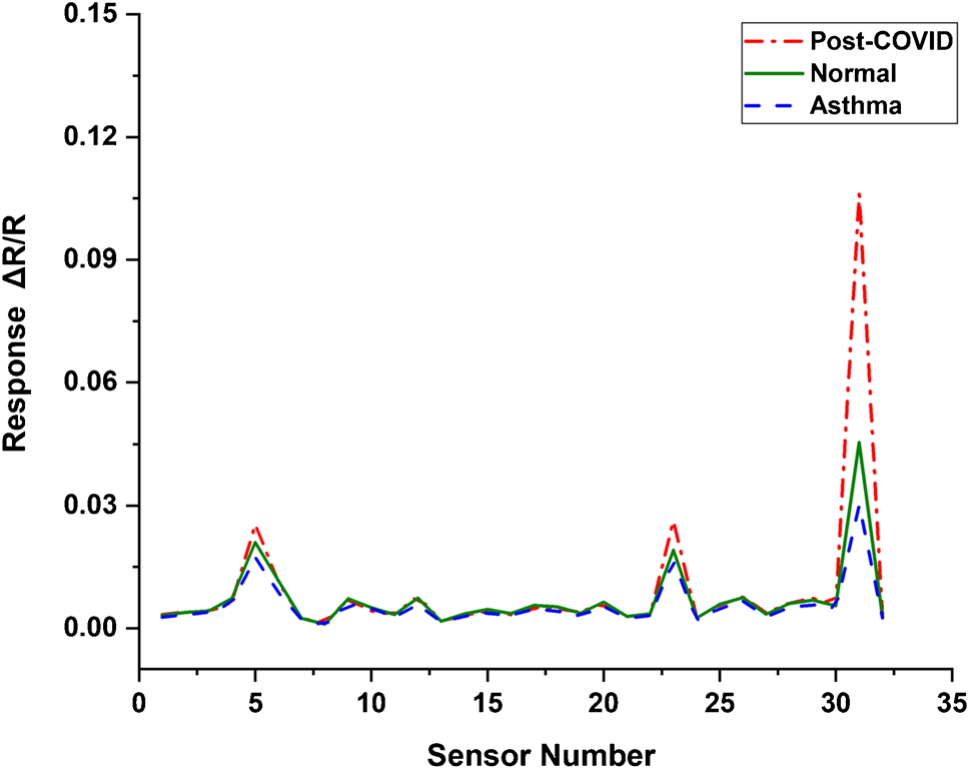
Sensor response of PCS, asthma, and normal breath samples from E-nose (Cyranose-320)

The sensors of the E-nose respond to the VOCs in the exhaled breath sample through a change Δ*R* in their resistance *R* values. The 32 sensor array consists of carbon black polymer composite sensors coated with different types of cross-reactive proprietary conducting polymers. C-320 sensors come with parts per million detection sensitivity ^21^. A recent study reported the C-320 sensor response to various VOCs. Table 2 represents the response of sensors 5, 23, and 31 with the standard VOC family. Figure 2 shows the average values of sensors’ response of the E-nose for PCS, asthma, and normal breath samples. The sensors behaved differently for PCS, asthma, and normal breath samples. It is seen that sensor numbers 5, 23, and 31 have shown noticeably different Δ*R*/*R* values for the three classes of samples.

**Table 2:** Sensor response of C-320 with some of the standard VOCs [ adapted from Doty AC et al. ^21^] (CC BY 4.0)

Sensor number	VOC	Sensor response
5	Aldehydes	Moderate
	Alcohol	Moderate
23	Aldehydes	Moderate
	Amines	Moderate
31	Ketones	Very high
	Amines	Moderate
	Aldehydes	Moderate
	Alcohol	Very high

**Fig. 3:**
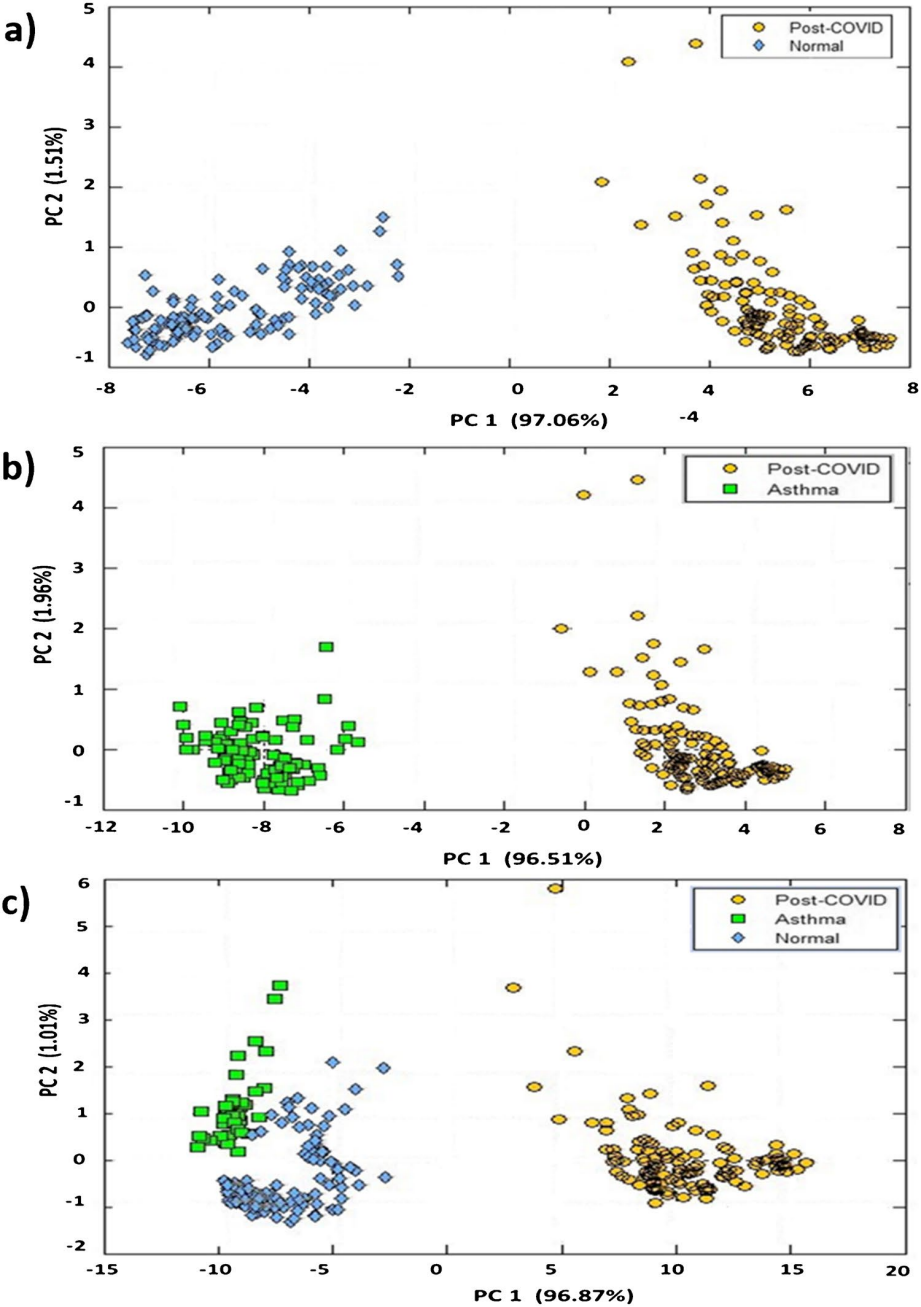
Score plot (PC1 vs. PC2) in PCA space with autoscale obtained from *k*-NN analysis of **a** PCS and normal, **b** PCS and asthma, and **c** PCS, asthma and normal

**Fig. 4:**
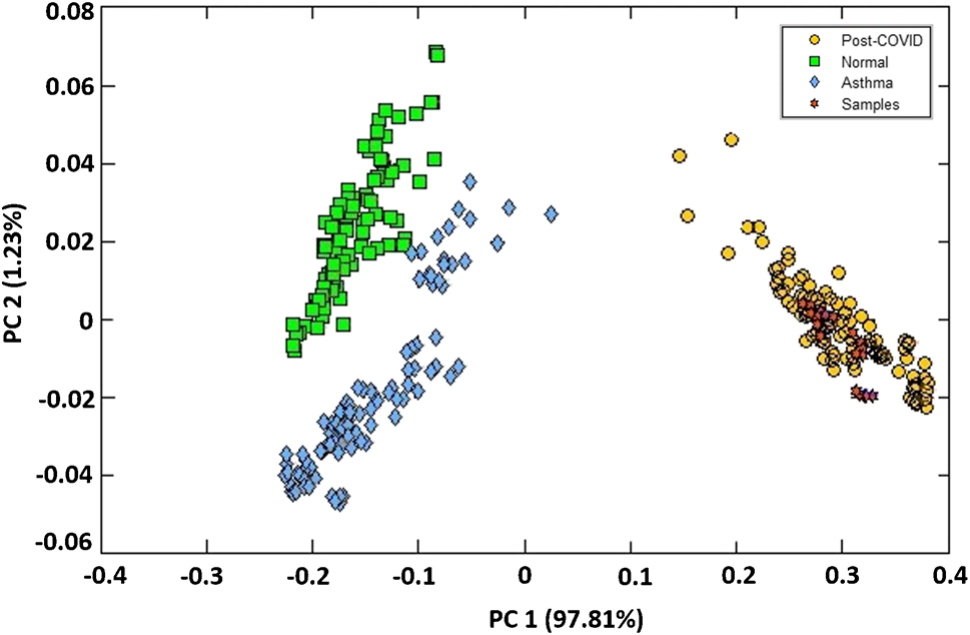
PCS samples predicted using standard model including normal, asthma, and PCS data. The data for prediction falls (dark brown stars) in the PCS cluster gives a very good prediction

**Fig. 5:**
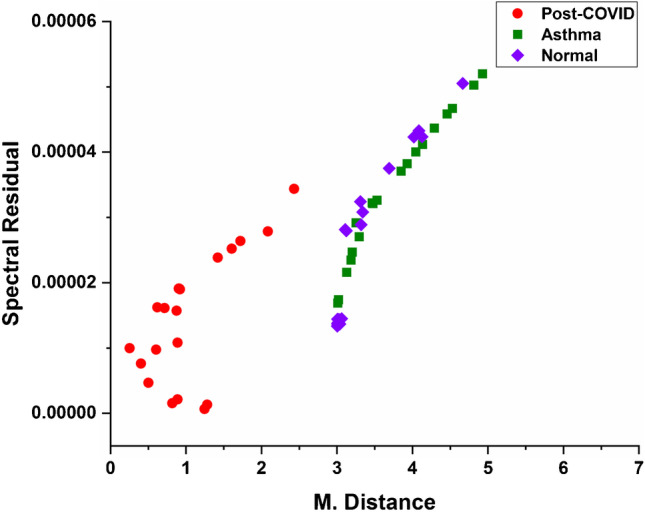
Plot of M. distance vs spectral residual obtained from match/no match from PCS set

Responses from all the 32 sensors have been taken to build the model. It is clear from Fig. 2 that sensor responses for all three classes (PCS, asthma, and control) are different. The *k*-NN regression analysis was carried out for the classification of PCS, asthma, and normal subjects, and the results are shown in Fig. 3. Figure 4 shows the prediction result of 20 PCS data using the *k*-NN model created. It is clearly seen from Fig. 4 that all the samples for which the condition was predicted clearly belong to the PCS standard calibration set, giving 100% accuracy. The match/no-match analysis output is shown in Table 3, and Fig. 5 represents the spectral residual vs. M. distance values of test samples used for match/no-match. From match/no-match test, the “sensitivity” and “specificity” of the breath analysis technique (E-nose) as applied to the PCS condition is calculated and is shown below.

**Table 3:** Match/no-match prediction report using PCS calibration set

Class	Sample nos. (trials)	Match	M. distance range	S. residual range
Normal	21–24 (5 each)	No	3.00–4.66	(1.33–5.04) × 10^–5^
Asthma	45–48 (5 each)	No	3.01–6.72	(1.68–7.32) × 10^–5^
Post-COVID	57–60 (5 each)	Yes	0.25–2.43	(0.0643–3.43) × 10^–5^

$$\mathrm{Sensitivity\;}=\mathrm{\;True\;Positive\;}/ (\mathrm{True\;Positive\;}+\mathrm{\;False\;Negative})$$

$$\mathrm{Sensitivity }=\mathrm{\;number\;of\;cases\;}"\mathrm{diagnosed}"\mathrm{\;as\;PCS}/\mathrm{\;number\;of\;}"\mathrm{actual}"\mathrm{\;PCS\;cases}$$

$$\mathrm{Sensitivity\;}= 20\;/\;(20+0) = 100\mathrm{\%}$$

$$\mathrm{Specificity\;}=\mathrm{\;True\;Negative\;}/ (\mathrm{True\;Negative\;}+\mathrm{\;False\;Positive})$$

$$\mathrm{Specificity\;}=\mathrm{\;number\;of\;cases\;}"\mathrm{diagnosed}"\mathrm{\;as\;not\;PCS\;}/ "\mathrm{actual}"\mathrm{\;number\;of\;cases\;that\;are\;not\;PCS}$$

$$\mathrm{Specificity\;}= 40\;/\;(40+0)\;=\;100\mathrm{\%}$$

**Fig. 6:**
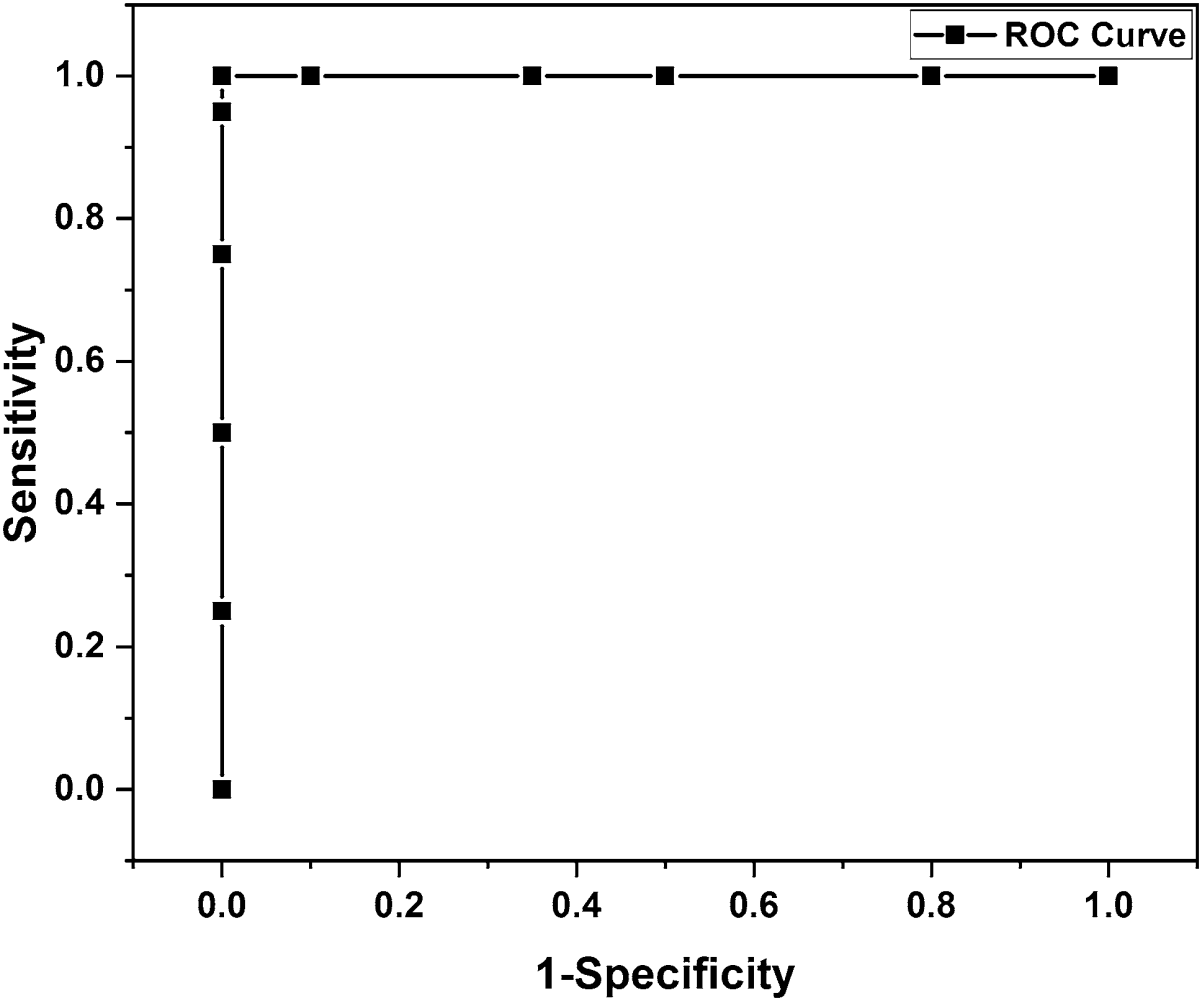
Receiver operating characteristic (ROC) curve of the present method for the screening of PCS

The diagnostic ability of this classification can be observed by plotting the ROC curve ^22^. The ROC curve was plotted using specificity and sensitivity values corresponding to selected cut-off thresholds for M. distance (Fig. 6). The area under the curve (AUC) of the ROC curve is obtained as 1.

## Discussion

A few groups have already studied the use of exhaled breath analysis for the diagnosis of COVID-19 [23–26]. At present, a number of commercial breath analysis devices are available in the market for COVID-19 diagnosis [26, 27]. Different groups have identified different VOC markers, such variations presumably arising due to the differences in operation principle of those techniques (GC–MS, advanced laser spectroscopy techniques, electronic nose, etc.) used for the measurements.

PCS patients experience several complications such as difficulties in breathing, cough, nausea, and muscle weakness, for long periods even after getting cured from COVID-19 infection. PCS can cause severe damages to the respiratory system and other parts of the body, which may even lead to life-threatening conditions. It has been observed that many of the symptoms are similar to that of COPD and asthma. Popular imaging modalities such as X-ray imaging and computational tomography (CT) have been found to be not able to provide a proper diagnosis, failing to show any difference for the PCS with respect to other respiratory disorders.

Because of the continued spread of the COVID-19 disease and the evolution of the PCS/MIS, MIS-C phenomena, the need for developing fast, easy, POC technique for monitoring, and early detection of PCS have become an urgent need at present. In view of the higher susceptibility of the population with co-morbidities like asthma and coronary problems to the PCS cases, it is very important that other similar conditions should not influence the method adopted for PCS screening. We have selected a cohort of asthma subjects to meet this requirement in the present study. Also, asthma and COPD have several common breath markers, and hence the results from our studies could be extrapolated to COPD cases also with a high degree of reliability. Breath analysis has the potential to detect VOC markers associated with many respiratory disorders, including COVID-19 as mentioned earlier [23–26].

The diagnosis of PCS condition and comparison of the same with other lung diseases having similar symptoms (leads to misinterpretation) are not discussed in the study by Zamora-Mendoza et al. [17]. Rather than discriminating PCS and normal, the classification of PCS from other lung diseases is a necessity in order to provide proper medication to patients. Also, *k*-NN is better than the canonical discriminant analysis and support vector machine, provided that the training data is much larger than the no. of features. We have achieved the maximum sensitivity and specificity of PCS vs. normal group, which is higher than that reported by Zamora-Mendoza et al. [17]. Improvement in the present result is because of the the supervision of the classification algorithms used in this study.

Sensors 5, 23, and 31 show significant differences among PCS, asthma, and normal which may be due to the presence of breath VOCs generated during the metabolic changes in the body. Other 29 sensors also show their responses to breath samples from different classes. One could see that sensors 5, 23, and 31 have noticeable responses to aldehydes, ketones, amines, and alcohols (Table 2). Breath analysis using E-nose followed by regression analysis can give fairly good information about the health condition. Our preliminary study, presented here, shows that breath analysis is highly suitable for PCS diagnosis. We observed a 100% success in PCS discrimination from normal and asthma conditions using the *k*-NN multivariate regression analysis (Fig. 3 and Fig. 4). Similarly, the match/no-match test derived out of principal component analysis also gives 100% sensitivity and specificity for the prediction of PCS samples. ROC curve and AUC-ROC curve show that this method could be successfully used for clinical application.

In conclusion, the breath analysis using the E-nose technique discussed here will help point-of-care PCS diagnosis, especially in screening and deciding whether long-term abnormal conditions observed in people with co-morbidities have arisen from other diseases or from PCS only, which may help in the planning of necessary therapy.

## Supplementary Information

Below is the link to the electronic supplementary material.

Supplementary file1 (PDF 103 KB)
