# Supplementary material for: Post-COVID syndrome screening through breath analysis using electronic nose technology
Source: Anal Bioanal Chem. 2022 Mar 18;414(12):3617–24. doi: 10.1007/s00216-022-03990-z (PMC8930465; doi:10.1007/s00216-022-03990-z)
Supplement: Supplementary file 1 — Supplementary file1 (PDF 103 KB) [file 216_2022_3990_MOESM1_ESM.pdf]

**Sample Number**	**Habits**<br>**(smoking,tobacco chewing,alcohol,)**	**Sex**	**Age**	**Clinical diagnosis**
1 to 24	None	10 F, 14M	21- 45	Healthy
25	None	F	65	PCS
26	Tobacco chewing	M	82	PCS
27	None	F	34	PCS
28	None	F	53	PCS
29	Smoking	M	35	PCS
30	None	F	20	PCS
31	None	M	20	PCS
32	None	M	25	PCS
33	None	M	25	PCS
34	None	F	62	PCS
35	None	F	63	PCS
36	Alcohol	M	58	PCS
37	None	F	66	PCS
38	None	M	61	PCS
39	Tobacco chewing	M	48	PCS
40	None	F	42	PCS
41	None	F	63	PCS
42	None	M	14	PCS
43	None	M	71	PCS
44	None	F	76	PCS
45	None	M	49	PCS
46	Tobacco chewing	M	33	PCS
47	None	F	62	PCS
48	None	F	41	PCS

49	None	F	43	Asthma
50	None	M	36	Asthma
51	None	M	68	Asthma
52	None	F	77	Asthma
53	None	F	55	Asthma
54	None	F	60	Asthma
55	Alcohol	M	70	Asthma
56	None	F	64	Asthma
57	None	M	42	Asthma
58	None	F	49	Asthma
59	None	M	67	Asthma
60	None	F	54	Asthma
